# New record and diet of the endangered Mau Son Spiny Frog (*Quasipaaacanthophora* Dubois and Ohler, 2009) in Vietnam

**DOI:** 10.3897/BDJ.13.e154204

**Published:** 2025-04-29

**Authors:** Vien Hong Thi Nguyen, Anh Van Pham, Truong Quang Nguyen, Tung Thanh Tran, Thomas Ziegler, Cuong The Pham

**Affiliations:** 1 Faculty of Resources and Environment, University of Sciences, Thai Nguyen University, Thai Nguyen, Vietnam Faculty of Resources and Environment, University of Sciences, Thai Nguyen University Thai Nguyen Vietnam; 2 Faculty of Environmental Sciences, University of Science, Vietnam National University, Hanoi, Vietnam Faculty of Environmental Sciences, University of Science, Vietnam National University Hanoi Vietnam; 3 Institute of Biology, Vietnam Academy of Science and Technology, Hanoi, Vietnam Institute of Biology, Vietnam Academy of Science and Technology Hanoi Vietnam; 4 Graduate University of Science and Technology, Vietnam Academy of Science and Technology, Hanoi, Vietnam Graduate University of Science and Technology, Vietnam Academy of Science and Technology Hanoi Vietnam; 5 Vinh Phuc College, Vinh Phuc, Vietnam Vinh Phuc College Vinh Phuc Vietnam; 6 Cologne Zoo, Cologne, Germany Cologne Zoo Cologne Germany; 7 Institute of Zoology, University of Cologne, Cologne, Germany Institute of Zoology, University of Cologne Cologne Germany

**Keywords:** Dong Son–Ky Thuong Nature Reserve, invertebrates, northern Vietnam, prey items, stomach contents

## Abstract

**Background:**

The Mau Son Spiny Frog *Quasipaaacanthophora* was originally described from Mau Son Mountain, Lang Son Province in 2009 and subsequently recorded in Bac Giang and Quang Ninh Provinces of Vietnam. The species was listed as Vulnerable in the IUCN Red List 2017 and as Endangered in the Vietnam Red Data Book 2024. However, knowledge about diet ecology of the species is virtually lacking.

**New information:**

Based on recent fieldwork in northern Vietnam, we herein report a new population of *Quasipaaacanthophora* from Dong Son–Ky Thuong Nature Reserve in Quang Ninh Province. In addition, we provide data on the diet of *Q.acanthophora*, based on stomach content analyses of 38 individuals (10 males, 17 females, 11 subadults). A total of 27 prey categories containing 446 items were found in the stomachs of *Q.acanthophora*. The dominant prey items of the species were Blattodea, Coleoptera (Other Coleoptera), Orthoptera (Gryllidae), Decapoda and Hymenoptera (Formicidae). The most important (IRI) groups amongst the prey of Q.acanthophora were Blattidae (32.67%), followed by Gryllidae (9.11%), other Coleoptera (6.57%), Decapoda (5.72%) and Formicidae (5.61%).

## Introduction

The genus *Quasipaa* Dubois, 1992 currently contains 13 recognised species with a restricted distribution in Asia, known from China through the Indochina Region and southwards to Thailand (Frost 2025). In Vietnam, six species were recognised, viz. *Quasipaaacanthophora* Dubois and Ohler, 2009, *Q.boulengeri* (Günther, 1889), *Q.delacouri* (Angel 1928), *Q.spinosa* (David, 1875), *Q.taoi* Pham, Hoang, Phan, Nguyen & Ziegler, 2022 and *Q.verrucospinosa* (Bourret 1937) (Frost 2025), of which, the Mau Son Spiny Frog *Quasipaaacanthophora* is currently known only from northern Vietnam (Frost 2025). The species was described from Mau Son Mountain, Lang Son Province and subsequently recorded in Tay Yen Tu Nature Reserve of Bac Giang Province and Hai Ha District of Quang Ninh Province (Hecht et al. 2013, Pham et al. 2020, Frost 2025). The species was evaluated as Vulnerable (VU) in the IUCN Red List (IUCN SSC Amphibian Specialist Group 2017) and listed as Endangered (EN) in the Vietnam Red Data Book 2024 (Pham and Nguyen 2024) due to ongoing over-harvest for food consumption and decline in the extent and quality of its habitat. In terms of natural history, the species is found along rocky streams in evergreen forests. Dietary ecology of the endangered species remains unknown. In this study, we record a new population of *Q.acanthophora* from Dong Son–Ky Thuong Nature Reserve in Quang Ninh Province and provide the first data on diet composition of this species on the basis of stomach content analysis of frogs collected in Lang Son, Bac Giang and Quang Ninh Provinces, northern Vietnam.

## Materials and methods

**Sampling**

Field surveys were conducted at three sites in north-eastern Vietnam: (1) Tay Yen Tu Nature Reserve, Bac Giang Province (21°09' – 21°23'N, 106°38' – 107°02'E, elevations: 350–800 m a.s.l.) between 2015 and 2024 (five males, 12 females, eight subadults); (2) Mau Son Mountain, Lang Son Province (21°48′ – 21°52′N, 106°54′ – 107°01′E, elevations: 700–1000 m a.s.l.) in 2018, 2024 (four males, four females, two submales) and (3) Dong Son–Ky Thuong Nature Reserve, Quang Ninh Province (21°05' – 21°12'N, 106°56' – 107°13'E, elevations: 150–700 m a.s.l.) in 2024, 2025 (one male, one female, one subadult). The surrounding habitat consisted of evergreen forest with a mix of larger, medium and small hardwoods, bamboo and shrubs.

Frogs were captured by hand in streams between 19:00 h and 23:00 h following the guidelines approved by the American Society of Ichthyologists and Herpetologists for animal care (Beaupre et al. 2004). A stomach-flushing technique was applied to obtain stomach contents without sacrificing them (Griffiths 1986, Leclerc and Courtois 1993, Solé et al. 2005). Prey items were preserved in 70% ethanol. Frogs were subsequently released at the collecting site after measurements of snout-vent length (SVL), head length (HL), head width (HW) and mouth width (MW) with a digital caliper to the nearest 0.1 mm, taken and measured weight (BM) using electronic scales to the nearest 0.1 g.

**Species identification**

For taxonomic identification, two individuals were collected for voucher specimens. After having been photographed in life, frogs were anaesthetised and euthanised in a closed vessel with a piece of cotton wool containing ethyl acetate (Simmons 2002), fixed in 85% ethanol and subsequently stored in 70% ethanol. Measurements were taken with a digital calliper to the nearest 0.1 mm. Abbreviations are as follows: a.s.l.: above sea level; terminology of morphological characters followed Pham et al. (2022): snout-vent length (from tip of snout to cloaca) (SVL); head length (a parallel line with the vertebral column from posterior margin of mandible to tip of snout) (HL); maximum head width (at rictus) (HW); rostral length (from anterior corner of orbit to tip of snout) (RL); distance from nostril to tip of snout (NS); distance from anterior corner of orbit to nostril (EN); internarial distance (distance between nostrils) (IND); interorbital distance (IOD); eye diameter (ED); maximum width of upper eyelid (UEW); distance between anterior margins of orbits (DAE); distance between posterior margins of orbits (DPE); distance from posterior margin of mandible to nostril (MN); distance from posterior margin of mandible to anterior margin of orbit (MFE); distance from posterior margin of mandible to posterior margin of eye (MBE); upper arm length (from axilla to elbow) (UAL); fore-arm length (from elbow to tip of third finger) (FAL); femur length (from vent to knee) (FeL); tibia length (from knee to tarsus) (TbL); maximum tibia width (TbW); foot length (from tarsus to tip of fourth toe) (TbW); inner metatarsal tubercle length (IMT). For webbing formula, we followed Glaw and Vences (2007). Sex was determined by secondary sexual characters and gonadal inspection.

Determination of species was based on morphology following Dubois and Ohler (2009) and Pham et al. (2020). We also sequenced two samples of *Quasipaaacanthophora* collected from Dong Son–Ky Thuong Nature Reserve, Quang Ninh Province. We used the protocols of Pham et al. (2022) for DNA extraction, amplification and sequencing. A fragment of 16S rRNA gene, approximately 570 bp was amplified and sequenced using a primer pair LR-N-13398 (5´-CGCCTGTTTACCAAAAACAT-3´; forward) and LR-J 12887 (5´-CCGGTCTGAACTCAGATCACGT-3´; reverse) (Simon et al. 1994). Sequences were compared with those from GenBank using Basic Local Alignment Search Tool (BLAST) searches.

**Stomach content analysis**

Prey items were identified under a microscope (Olympus SZX7), based on identification keys (i.e. Naumann et al. (1991), Thai (2003), Johnson and Triplehorn (2005), Brusca et al. (2016)). The maximum length (L) and width (W) of each prey item were measured to the nearest 0.1 mm using either a calliper or a calibrated ocular micrometre fitted to a microscope. Body parts of the same individual were assembled before taking measurements, otherwise incomplete-bodied prey was measured separately. The volume (V) of prey item was calculated using the formula for a prolate spheroid (π = 3.14; Pham et al. (2022)): V = 4π/3*(L/2)*(W/2)^2.

To evaluate the relative importance of each prey category, we calculated the following three indices: %F, the frequency of occurrence (the percentage of stomachs containing specific prey categories amongst stomachs containing prey categories); %N, the relative number (the percentage of a specific prey categories amongst the number of the bulk of prey categories); and %V, the relative volume (the percentage of the volume of a specific prey categories amongst the volume of the bulk of prey categories (Nakamura and Tominaga 2021).

The index of relative importance (IRI) was used to determine the importance of each food category. This index provides a more informed estimation of prey item consumption than any of the three components alone by using the following formula (Caldart et al. 2012): IRI = (%F + %N + %V)/3,

where F is the frequency of prey occurrence in stomachs and N is the total number of prey items concerning all prey items. Statistical analyses were performed with the SPSS 20.0 (SPSS Inc., Chicago, Illinois, USA) and with the significance level set to p < 0.05 for all analyses. Data are presented as mean ± standard deviation (SD) unless otherwise noted. We used Kendall’s tau b statistics to examine the relationship between SVL and the prey volume. In addition, we also used Pearson’s correlations between the morphological measurements.

## Taxon treatments

### Quasipaa acanthophora

Dubois and Ohler, 2009

DF5A094D-0E97-5133-9E7A-344D5D7913B2

#### Materials

- **Type status:** Other material. **Occurrence:** catalogNumber: IEBR A.6385; individualCount: 1; sex: male; lifeStage: adult; occurrenceID: 9786FF3A-6233-5445-8488-A95B369871F1; **Taxon:** scientificName: *Quasipaaacanthophora*; class: Amphibia; order: Anura; family: Dicroglossidae; genus: Quasipaa; specificEpithet: acanthophora; scientificNameAuthorship: Dubois and Ohler, 2009; **Location:** country: Vietnam; countryCode: VN; stateProvince: Quang Ninh; locality: Dong Son-Ky Thuong Nature Reserve; verbatimElevation: 537 m; verbatimLatitude: 21°7.404'N; verbatimLongitude: 107°8.706'E; verbatimCoordinateSystem: WGS84; **Event:** eventDate: 16 March 2024; eventRemarks: collected by Ngo N.H, Do Q.H., and Nguyen N.T.; **Record Level:** language: en; collectionCode: Amphibians; basisOfRecord: PreservedSpecimen
- **Type status:** Other material. **Occurrence:** catalogNumber: IEBR A.6386; individualCount: 1; sex: female; lifeStage: adult; occurrenceID: 404F9789-AD42-59AA-8123-569FB9D50308; **Taxon:** scientificName: *Quasipaaacanthophora*; class: Amphibia; order: Anura; family: Dicroglossidae; genus: Quasipaa; specificEpithet: acanthophora; scientificNameAuthorship: Dubois and Ohler, 2009; **Location:** country: Vietnam; countryCode: VN; stateProvince: Quang Ninh; locality: Dong Son-Ky Thuong Nature Reserve; verbatimElevation: 640 m; verbatimLatitude: 21°7.410'N; verbatimLongitude: 107°8.706'E; verbatimCoordinateSystem: WGS84; **Event:** eventDate: 16 March 2024; eventRemarks: collected by Ngo N.H, Do Q.H., and Nguyen N.T.; **Record Level:** language: en; collectionCode: Amphibians; basisOfRecord: PreservedSpecimen

#### Description

Two sequences (GenBank accession numbers PV541279 and PV541280) of *Quasipaa* specimens from Quang Ninh Province were similar (99.2% and 99.5%, respectively) to the available sequence of *Q.acanthophora* (accession number OP326694 from type locality, Mau Son Mountain, Lang Son Province) in GenBank.

**Figure 1.**
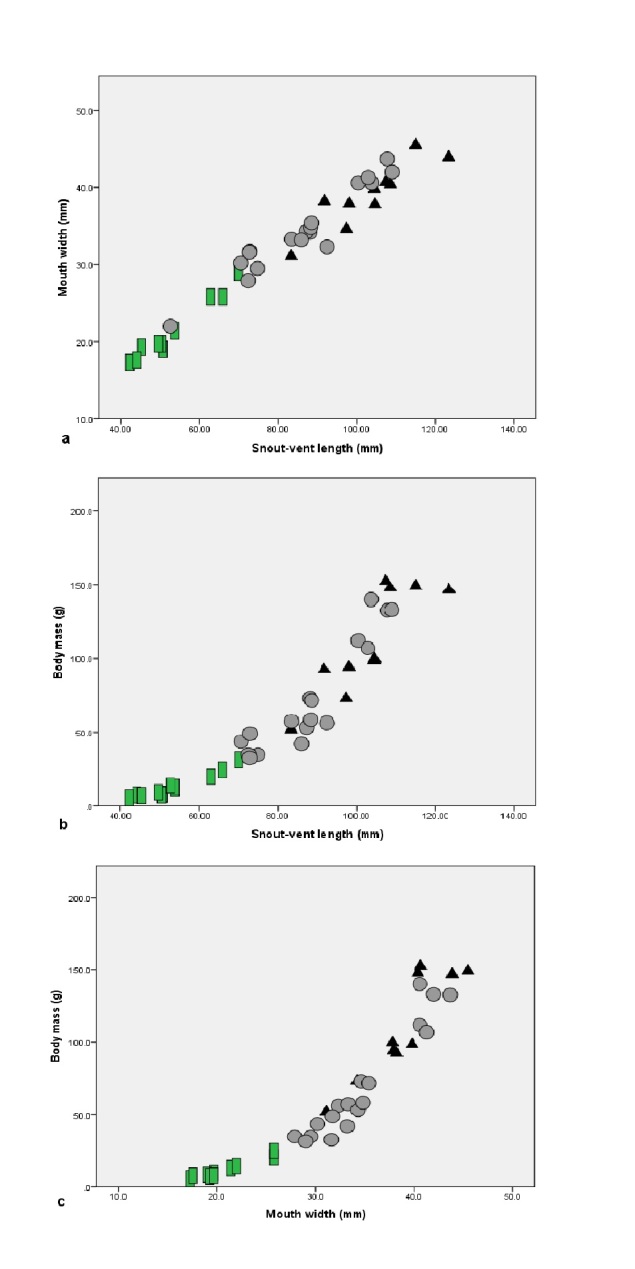
Dispersion diagrams from Pearson’s correlations between snout-vent length and mouth width (a), snout-vent length and body mass (b) and mouth width and body mass (c) of *Quasipaaacanthophora* in Vietnam. Triangles: Males; dots: Females; rectangles: Subadults.

Morphological characters of the specimens from Bac Giang, Lang Son and Quang Ninh Provinces agreed well with the descriptions of Dubois and Ohler (2009) and Pham et al. (2020): Size large, SVL 83.32–123.40 mm (103.41 ± 11.46 mm, n = 10), MW 31.10–45.50 mm (38.99 ± 4.17 mm, n = 10) and body mass (BM): 51.90–152.40 g, 110.52 ± 35.97 g, n = 10) in males; SVL 70.5–109.00 mm; 88.29 ± 12.97 mm, n = 17), MW 27.90–43.70 mm (35.09 ± 4.79 mm, n = 17) and body mass (BM 32.40–140.00 g, 73.39 ± 37.44 g, n = 17) in females; and SVL 42.3–69.93 mm (53.40 ± 9.08 mm, n = 11), MW 17.3–29.0 mm (21.51 ± 3.79 mm, n = 11) and body mass (BM): 5.3–31.3 g, 13.23 ± 8.46 g, n = 11) in subadults. There were strong positive correlations between the morphological measurements (SVL and MW: r = 0.981, p < 0.001; SVL and BM: r = 0.940; p < 0.001; MW and BM: r = 0.936, p < 0.001) (Fig. 1).

Morphological characteristics of two individuals collected from Dong Son–Ky Thuong Nature Reserve, Quang Ninh Province for taxonomic identification: A large frog, habitus robust with enlarged head (HL 37.4 mm, HW 39.8 mm in male and HL 38.4 mm, HW 42.8 mm in female); snout round anteriorly in dorsal view, projecting beyond lower jaw; rostral length greater than eye diameter (RL 12.3 mm, ED 11.5 mm in male and RL 12.9 mm, ED 12.7 mm in female); nostrils oval, closer to eye than to the tip of snout (NS 6.9 mm, EN 5.7 mm in male and NS 6.7 mm, EN 6.3 mm in female); internarial distance wider than interorbital distance and upper eyelid width (IND 9.3 mm, IOD 7.5 mm, UEW 7.7 mm in male and IND 9.2 mm, IOD 8.1 mm, UEW 7.9 mm in female) (Table 1); tympanum indistinct; vomerine teeth in two oblique ridges; tongue cordiform, notched posteriorly; external vocal sac absent.

Fore-limb: arms short; upper arm length (UAL 22.4 mm in male, 21.6 in female), forearm length (FAL 46.2 mm in male and FAL 47.7 mm in female); relative finger lengths: II < I < IV < III; fingers free of webbing; sides of fingers II and III with narrow dermal ridge; tips of fingers swollen, not expanded; subarticular tubercles prominent, round, formula 1, 1, 2, 2; inner metatarsal tubercle round; outer metatarsal tubercle elongate; Finger I with nuptial pad in male (Table 1).

Hind-limb: tibia length longer than thigh length (FeL 46.6 mm, TbL 50.1 mm in male and FeL 49.8 mm, TbL 51.7 mm in female); tips of toes swollen, round; relative length of toes: I < II < V< III < IV; toes fully webbed to distal end of terminal phalanx; dermal ridge present on outer sides of toes I and V; subarticular tubercles prominent, oval, formula 1, 1, 2, 3, 2; inner metatarsal tubercle elongate; outer metatarsal tubercle absent (Table 1).

**Figure 2.**
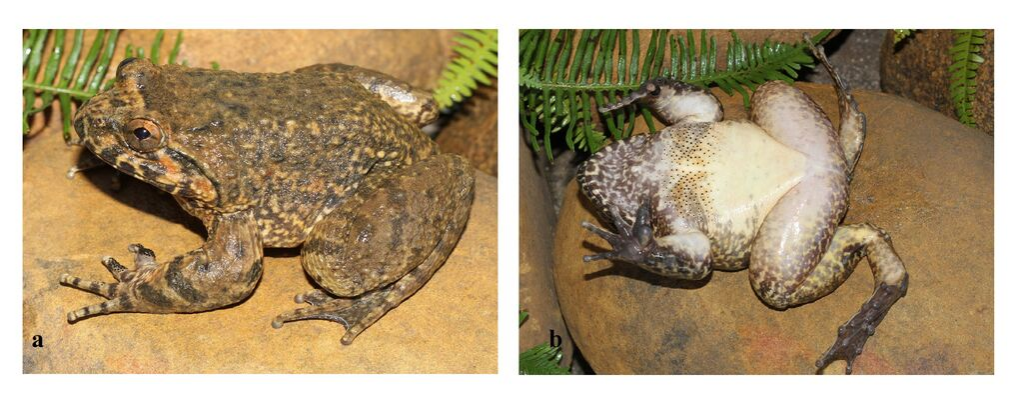
*Quasipaaacanthophora* (male, IEBR A.6385) from Dong Son–Ky Thuong Nature Reserve, Quang Ninh Province, Vietnam: **a** dorsal view; **b** ventral view.

**Figure 3.**
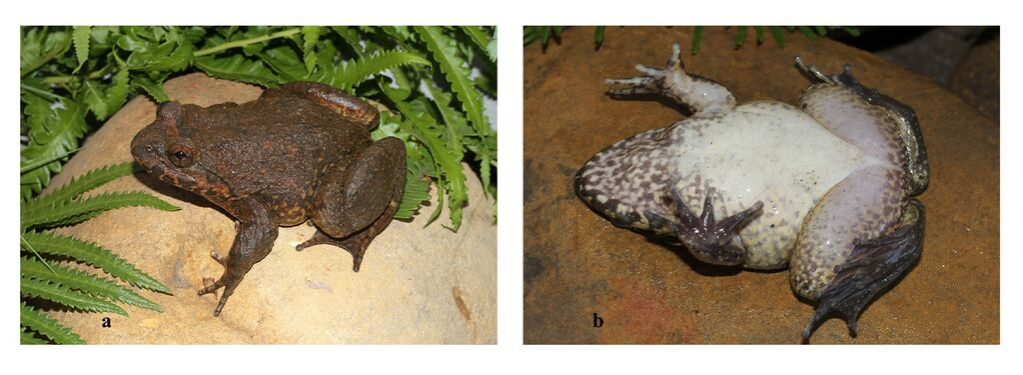
*Quasipaaacanthophora* (female, IEBR A.6386) from Dong Son–Ky Thuong Nature Reserve, Quang Ninh Province, Vietnam: **a** dorsal view; **b** ventral view.

Skin texture in life. Dorsal skin shagreened with regularly disposed glandular warts on back; upper part of flanks shagreened with elongated glandular warts; supratympanic fold prominent, from eye to above arm; belly and ventral surface of thigh smooth. Male with nuptial spines present on prepollex and Finger I (two separate pads), fingers II and III and chest (Figs 2, 3).

Colouration in life. Iris pale copper, dorsum and upper part of flanks brown; lower part of flanks light brown with whitish-yellow marbling; throat and chest brown with whitish marblings; dorsal surface of limbs brown with dark crossbars; belly immaculate white; toe webbing dark brown (Figs 2, 3).

#### Distribution

In Vietnam, this species was recorded in three provinces: Lang Son (Mau Son Mountain), Bac Giang (Tay Yen Tu Nature Reserve) and Quang Ninh (Hai Ha District) (Dubois and Ohler 2009, Hecht et al. 2013, Pham et al. 2020). This is the first record of the species from Dong Son–Ky Thuong Nature Reserve in Quang Ninh Province.

#### Ecology

In Dong Son–Ky Thuong Nature Reserve, frogs were observed between 19:00 h and 23:00 h in the headwaters of rocky streams. They were found in the water or on the ground of stream banks at elevations between 150 and 700 m a.s.l. The surrounding habitat was secondary forest of large, medium and small hardwoods mixed with shrubs and vines. Air temperatures at the sites ranged from 24.2–29.8°C and relative humidity was 72–86%.

#### Diet

For stomach flushing, 38 individuals (10 males, 17 females and 11 subadults) of *Q.acanthophora* were collected in Lang Son, Bac Giang and Quang Ninh Provinces. We identified 446 prey items of *Q.acanthophora*, including 138 prey items in males, 198 prey items in females and 110 prey items in subadults.

The number of prey items per individual was 1-29 items (average 11.74 ± 7.19 items, n = 38). Mean prey item length was 7.92 ± 7.84 mm (ranging from 1.80 to 85.00 mm, n = 446) and mean prey item width was 3.25 ± 2.69 mm (ranging from 0.50 to 30.00 mm, n = 446). The average volume per individual was 265.74 ± 616.03 mm^3^ (ranging from 7.06 to 3,286.31 mm^3^, n = 38) (Table 2).

**Figure 4.**
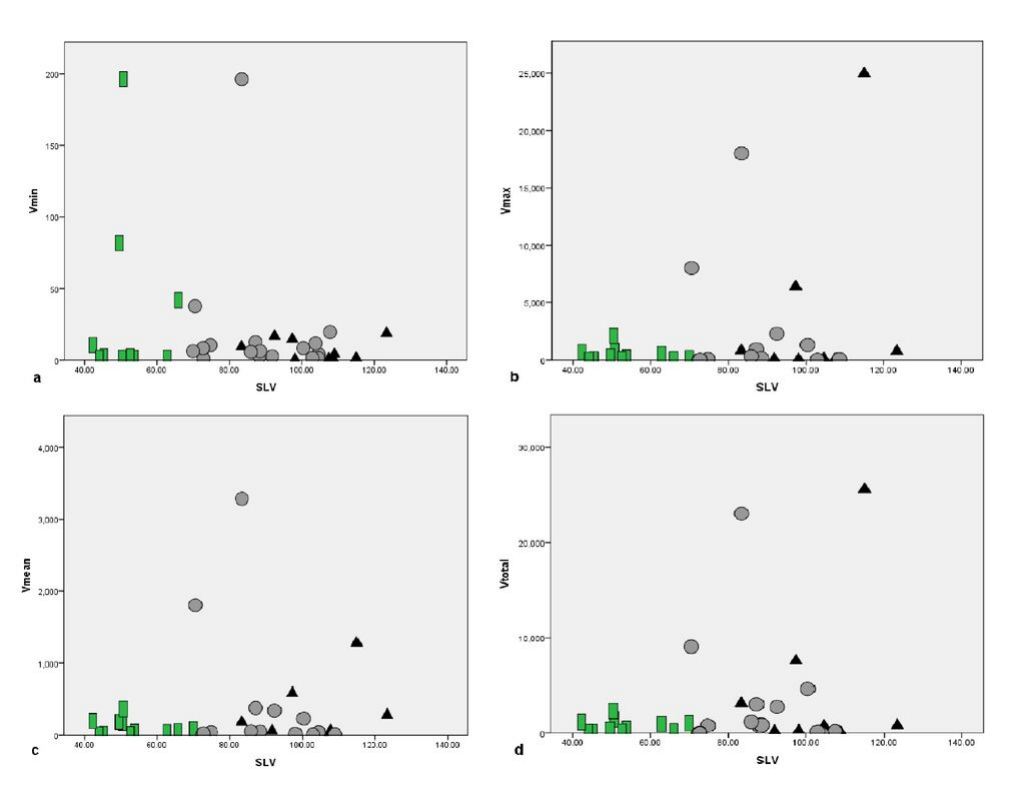
Relationships between the frog SVL (mm) and the minimum (a), maximum (b) and the mean (c) prey item volume and the total prey volume (d) of *Quasipaaacanthophora* in Vietnam. Triangles: Males; dots: Females; rectangles: Subadults; Vmin = minimum prey item volume (mm); Vmax = maximum prey item volume (mm); Vmean = mean prey item volume (mm); Vtotal = the total prey volume (mm).

There was no positive correlation between the frog SVL and the minimum prey volume (Kendall’s tau b: tau = -0.085, P = 0.500), mean prey item volume (tau = -0.024, P = 0.839), maximum prey item volume (tau = -0.028, P = 0.808) and the total prey volume (tau = -0.04, P= 0.971) (Fig. 4).

We identified 27 categories in the stomachs of *Q.acanthophora*. Insects are the main food component of *Q.acanthophora*, with 21 categories (Blattidae, Carabidae, Elateridae, Hybosoridae, Hydraenidae, Scarabaeidae, Chrysomelidae, Lucanidae, Meloidae, Hydrophilidae, larvae of Coleoptera, other Coleoptera, Forficulidae, Tipulidae, Pentatomidae, Formicidae, Termitidae, Acrididae, Gryllidae, Tettigoniidae, Phasmatodea, Diapheromeridae) and other invertebrates (Myriapoda, Uropygi, Decapoda and Oligochaeta) (Table 3). The most number of prey items was Blattidae (46.64%, n = 446), followed by other Coleoptera (8.97%), Acrididae (5.61%) and Formicidae (4.93%), while the most frequently foraged prey group was also Blattidae (24.77%), followed by Formicidae (11.01%), other Coleoptera (10.09%), Elateridae and larvae Coleoptera (7.34%). In the comparisons by the IRI, Blattidae (32.67%), followed by Gryllidae (9.11 %), other Coleoptera (6.57%), Decapoda (5.72%) and Formicidae (5.61%) were evaluated as the most important diet groups of *Q.acanthophora* (Table 3).

## Discussion

The diet of *Quasipaaacanthophora* mainly consisted of insects with nine orders (Blattodea, Coleoptera, Dermaptera, Diptera, Hemiptera, Hymenoptera, Isoptera, Orthoptera and Phasmatodea), which included 21 prey categories. Of which, Blattodea, Coleoptera, Orthoptera and Hymenoptera were the most important prey categories (IRI > 5%). Insects are also common food for some other amphibian species (viz. *Quasipaaverrucospinosa*, *Nanoranayunnanensis*, *Odorranachapaensis*, *O.morafkai*, *Microhylabutleri* and *M.heymonsi*, *Polypedatesmegacephalus*, *Occidozygamartensii*) in Vietnam (Ngo et al. 2014, Pham and Nguyen 2018, Pham et al. 2019, Le et al. 2020, Pham et al. 2022, Pham et al. 2023, Pham et al. 2024). These are terrestrial prey and are commonly found in the species' natural habitat (Pham et al. 2019, Sonephet et al. 2023, Pham et al. 2023). Other invertebrate groups, for example Decapoda, Myriapoda (Chilopoda, Diplopoda), Oligochaeta and Uropygi (Thelyphonidae) were also found in the stomach contents of the *Q.acanthophora*.

Ngo et al. (2014) reported the dietary composition of *Quasipaaverrucospinosa* from Central Vietnam. Both *Q.acanthophora* and *Q.verrucospinosa* occupy similar habitats, viz. the streams in evergreen forests with hardwoods, bamboo and shrub (Ngo et al. 2014, Pham et al. 2020). Their diet compositions are similar to each other with prey items of Diplopoda, Chilopoda, Blattodea, Coleoptera, Dermaptera, Isoptera, Hemiptera, Hymenoptera, Orthoptera, Insect larvae, Phasmatodea, Diptera and Decapoda (Ngo et al. 2014). Nonetheless, Uropygi and Oligochaeta were found exclusively in the diet of *Q.acanthophora*, whereas Araneae, Ephemeroptera, Odonata, Isopoda, Amphibia (Anura), Collemboda, Neuroptera, Lumbriculida, Megadrili, Gastropoda (Mesogastropoda), Cypriniformes and Perciformes occurred only in the diet of *Q.verrucospinosa* (Ngo et al. 2014).

**Figure 5.**
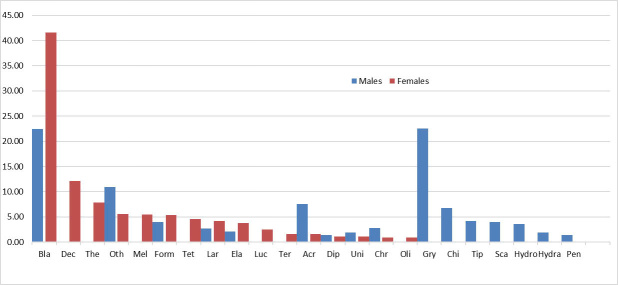
Importance indices (IRI) for prey categories consumed by males (blue) vs. females (red) of *Quasipaaacanthophora* in Vietnam. Acr = Acrididae, Bla = Blattidae, Car = Carabidae, Chi = Chilopoda, Chr = Chrysomelidae, Dec = Decapoda, Dia = Diapheromeridae, Dip = Diplopoda, Ela = Elateridae, Forf = Forficulidae, Form = Formicidae, Gry = Gryllidae, Hyb = Hybosoridae, Hydra = Hydraenidae, Hydro = Hydrophilidae, Lar = Larvae Coleoptera, Luc = Lucanidae, Mel = Meloidae, Oli = Oligochaeta, Oth = Other Coleoptera, Pen = Pentatomidae, Sca = Scarabaeidae, Ter = Termitidae, Tet = Tettigoniidae, Tip = Tipulidae, The = Thelyphonidae.

Both sexes had a diverse prey spectrum, comprising Blattidae, other Coleoptera, Formicidae, larvae Coleoptera, Elateridae, Acrididae, Diplopoda and Chrysomelidae. Gryllidae, Scarabaeidae, Pentatomidae, Tipulidae, Chilopoda, Hydraenidae and Hydrophilidae were found only in the stomachs of males, while Decapoda, Thelyphonidae, Meloidae, Tettigoniidae, Termitidae, Oligochaeta and Lucanidae were found only in the stomachs of females. Blattodea and other Coleoptera were the most important foods of both sexes (IRI > 5%) (Fig. 5). Blattodea was the most important food of both adults and subadults (IRI > 5%). The findings need to be confirmed in more in-depth studies with larger sample size.

## Supplementary Material

XML Treatment for Quasipaa acanthophora

## Figures and Tables

**Table 1.** Measurements (in mm) of *Quasipaaacanthophora* collected from Dong Son–Ky Thuong Nature Reserve, Quang Ninh Province, Vietnam.

	IEBR A.6385	IEBR A.6386	Dubois and Ohler (2009)
Sex	Male	Female	Male (holotype)
SVL	98.1	100.4	101.7
HL	37.4	38.4	38.5
HW	39.8	42.8	41.0
MN	31.8	35.7	32.2
MFE	27.7	29,5	25,1
MBE	18.6	19.4	17.2
RL	12.3	12.9	14.4
ED	11.5	12.7	11.2
UEW	7.7	7.9	9.0
IND	9.3	9.2	10.0
IOD	7.5	8.1	8.1
DAE	15.5	15.4	15.3
DPE	27.2	27.1	26.8
NS	6.9	6.7	7.8
EN	5.7	6.3	6.8
UAL	22.4	21.6	25.9
FAL	46.1	47.7	45.2
FeL	46.6	49.8	52.8
TbL	50.1	51.7	53.5
TbW	20.7	21.0	18.8
FoL	64.8	72.3	72.1
IMT	8.2	9.0	8.3
HL/SVL	0.38	0.38	0.38
HW/SVL	0.41	0.43	0.40
RL/SVL	0.13	0.13	0.14
HL/HW	0.94	0.90	0.94
TbL/SVL	0.51	0.51	0.53
TbL/TbW	2.42	2.46	2.85
MFE/MBE	1.49	1.52	1.46
DAE/DBE	0.57	0.57	0.57

**Table 2.** Summary (Total, Mean, SD and range) of the width (W), length (L), volume (V) and prey item number (N) data for *Quasipaaacanthophora* in Vietnam (in mm for W and L; in mm^3^ for V).

	W	L	Prey item volume				N
			Minimum	Maximum	Mean	Total	
Total (n = 38)	3.25±2.69	7.92±7.84	19.95±44.72	1846.71±5034.34	265.74±616.03	2508.52±5562.11	11.74±7.19
	0.5–30	1.8–85	0.39–196.25	11.78–24963	7.06–3286.31	11.78–25509.45	1–29
Males (n = 10)	3.14±3.02	7.18±8.75	5.53±6.53	3358.63±7836.84	250.58±402.3	3929.6±7923.39	13.8±5.9
	0.5–30	1.8–85	0.39–18.84	52.99–24963	12.36–1275.47	185.46–25509.45	3–20
Females (n = 17)	3.58±2.88	8.57±6.96	20.87±46.03	1859.43±4590.24	376.33±865.26	2797.68±5700.89	11.65±8.5
	1–25	2–55	1.05–196.25	11.78–17989.58	7.06–3286.31	11.78–23004.16	1–29
Subadult (n = 11)	2.82±1.66	7.69±8.08	31.92±55.1	879.09±1302.95	187.4±239.37	1305.07±1567.34	10.01±5.56
	1–10	2–60	1.47–196.25	23.55–4590.24	9.95–865.26	149.22–5700.89	2–23

**Table 3.** Prey categories consumed by *Quasipaaacanthophora* in Vietnam (n = 38), (F) total frequency, (%F) relative frequency, (N) total abundance, (%N) relative abundance, (V) total volume (mm³), (%V) relative volume; (IRI) importance index.

Prey taxa	F	%F	N	%N	V	%V	IRI
**Decapoda**	**4.00**	**3.67**	**16.00**	**3.59**	**9429.03**	**9.89**	**5.72**
**Myriapoda**	**5.00**	**4.59**	**6.00**	**1.35**	**7343.36**	**7.70**	**4.55**
Chilopoda	2.00	1.83	2.00	0.45	7190.60	7.54	3.28
Diplopoda	3.00	2.75	4.00	0.90	152.76	0.16	1.27
**Oligochaeta**	**3.00**	**2.75**	**6.00**	**1.35**	**1066.95**	**1.12**	**1.74**
**Uropygi**	**1.00**	**0.92**	**5.00**	**1.12**	**9022.79**	**9.47**	**3.83**
Thelyphonidae	1.00	0.92	5.00	1.12	9022.79	9.47	3.83
**Blattodea**	**27.00**	**24.77**	**208.00**	**46.64**	**25352.07**	**26.60**	**32.67**
Blattidae	27.00	24.77	208.00	46.64	25352.07	26.60	32.67
**Coleoptera**	**41.00**	**37.61**	**114.00**	**25.56**	**8992.62**	**9.43**	**24.20**
Carabidae	2.00	1.83	5.00	1.12	834.09	0.88	1.28
Elateridae	8.00	7.34	14.00	3.14	353.41	0.37	3.62
Hybosoridae	1.00	0.92	3.00	0.67	27.74	0.03	0.54
Hydraenidae	2.00	1.83	7.00	1.57	690.93	0.72	1.38
Scarabaeidae	2.00	1.83	2.00	0.45	1610.75	1.69	1.32
Chrysomelidae	3.00	2.75	3.00	0.67	200.96	0.21	1.21
Lucanidae	1.00	0.92	1.00	0.22	2289.58	2.40	1.18
Meloidae	2.00	1.83	18.00	4.04	1565.42	1.64	2.50
Hydrophilidae	1.00	0.92	9.00	2.02	389.10	0.41	1.11
Larvae	8.00	7.34	12.00	2.69	398.65	0.42	3.48
Other Coleoptera	11.00	10.09	40.00	8.97	631.99	0.66	6.57
**Dermaptera**	**1.00**	**0.92**	**2.00**	**0.45**	**136.59**	**0.14**	**0.50**
Forficulidae	1.00	0.92	2.00	0.45	136.59	0.14	0.50
**Diptera**	**2.00**	**1.83**	**14.00**	**3.14**	**2364.55**	**2.48**	**2.48**
Tipulidae	2.00	1.83	14.00	3.14	2364.55	2.48	2.48
**Hemiptera**	**2.00**	**1.83**	**2.00**	**0.45**	**376.80**	**0.40**	**0.89**
Pentatomidae	2.00	1.83	2.00	0.45	376.80	0.40	0.89
**Hymenoptera**	**12.00**	**11.01**	**22.00**	**4.93**	**839.03**	**0.88**	**5.61**
Formicidae	12.00	11.01	22.00	4.93	839.03	0.88	5.61
**Isoptera**	**2.00**	**1.83**	**8.00**	**1.79**	**603.18**	**0.63**	**1.42**
Termitidae	2.00	1.83	8.00	1.79	603.18	0.63	1.42
**Orthoptera**	**6.00**	**5.50**	**37.00**	**8.30**	**27541.33**	**28.89**	**14.23**
Acrididae	3.00	2.75	25.00	5.61	706.11	0.74	3.03
Gryllidae	1.00	0.92	1.00	0.22	24963.00	26.19	9.11
Tettigoniidae	2.00	1.83	11.00	2.47	1872.23	1.96	2.09
**Phasmatodea**	**1.00**	**0.92**	**1.00**	**0.22**	**2093.33**	**2.20**	**1.11**
Diapheromeridae	1.00	0.92	1.00	0.22	2093.33	2.20	1.11
**Unidentified**	**2.00**	**1.83**	**5.00**	**1.12**	**162.23**	**0.17**	**1.04**
